# Efficiency of New Dose Escalation Designs in Dose-Finding Phase I Trials of Molecularly Targeted Agents

**DOI:** 10.1371/journal.pone.0051039

**Published:** 2012-12-12

**Authors:** Christophe Le Tourneau, Hui K. Gan, Albiruni R. A. Razak, Xavier Paoletti

**Affiliations:** 1 Department of Medical Oncology, Institut Curie, Paris, France; 2 Joint Austin-Ludwig Oncology Unit, Austin Hospital, Melbourne, Australia; 3 Department of Medical Oncology, Princess Margaret Hospital, Toronto, Canada; 4 Department of Biostatistics, Institut Curie, Paris, France; 5 Institut Curie / INSERM U900, Paris, France; Davidoff Center, Israel

## Abstract

**Background:**

Statistical simulations have consistently demonstrated that new dose-escalation designs such as accelerated titration design (ATD) and continual reassessment method (CRM)-type designs outperform the standard “3+3” design in phase I cancer clinical trials.

**Methods:**

We evaluated the actual efficiency of different dose escalation methods employed in first-in-human phase I clinical trials of targeted agents administered as single agents published over the last decade.

**Results:**

Forty-nine per cent of the 84 retrieved trials used the standard “3+3” design. Newer designs used included ATD in 42%, modified CRM [mCRM] in 7%, and pharmacologically guided dose escalation in 1%. The median numbers of dose levels explored in trials using “3+3”, ATD and mCRM designs were 6, 8 and 10, respectively. More strikingly, the mean MTD to starting dose ratio appeared to be at least twice as high for trials using mCRM or ATD designs as for trials using a standard “3+3” design. Despite this, the mean number of patients exposed to a dose below the MTD was similar in trials using “3+3”, ATD and mCRM designs.

**Conclusion:**

Our results support a more extensive implementation of innovative dose escalation designs such as mCRM and ATD in phase I cancer clinical trials of molecularly targeted agents.

## Introduction

The primary goal of phase I cancer clinical trials is to identify the dose to recommend for further evaluation (the recommended phase II dose [RP2D]), while exposing as few patients as possible to potentially sub-therapeutic or intolerable doses. In oncology, the RP2D is usually the highest dose with acceptable toxicity, usually defined as the dose level producing around 20% of dose-limiting toxicity. In North America, the Maximum Tolerated Dose (MTD) is the RP2D, whereas in the rest of the world, the MTD is considered the dose level above the RP2D.

Several dose escalation methods have been developed over time to best determine the RP2D. The standard method is the algorithm-based “3+3” design. Newer algorithm-based methods include the accelerated titration designs (ATD) [1]. There are also model-based designs such as the continual reassessment method (CRM) and its modifications [1]. The standard “3+3” design remains the most commonly used dose escalation design in phase I oncology trials [1], [2]. The statistical literature commonly evaluates the efficiency of dose escalation methods used in phase I trials by simulating the efficiency of these designs measuring specific parameters such as the accuracy of the established MTD, the absolute number of patients exposed at potentially sub-therapeutic doses below the MTD, the absolute number of patients experiencing severe toxicity and the trial duration. Overall, these statistical simulations have consistently come to the conclusion that the CRM-type designs outperform the standard “3+3” design [3]. A couple of studies have compared the actual efficiency of the CRM *versus* the standard “3+3” design in the published literature [4], [5]. However, these studies are of less relevance today as they were reported prior to the explosive developmental era of molecularly targeted therapy. Furthermore, results were conflicting in terms of trial duration and number of patients exposed at doses below the MTD, making it difficult to draw any firm conclusions.

## Patients and Methods

This study is a literature review that did not directly involved patients and was therefore not submitted to an ethics committee. In order to get more insight on the efficiency of new dose escalation methods in phase I trials of molecularly targeted agents, design information from 84 trials that reached the MTD over the last decade was abstracted. Molecularly targeted agents were defined in our study as anticancer agents that selectively target molecular pathways, as opposed to DNA, tubulin or cell division machinery. Only trials administering molecularly targeted agents orally or intravenously were included. Hormonal therapy and biological therapeutics such as immunotherapy, gene therapy and vaccines were excluded because of their unique mechanisms of action and toxicities. Trials were also excluded if they were testing drug combinations, reported only in abstract format or in languages other than English. To comprehensively identify phase I trials of molecularly targeted agents, we searched SCOPUS database from January 1st, 2000 to April 18th, 2010 [6].

While accuracy of the RP2D could not be evaluated, we compared the different dose-escalation designs on the other efficiency parameters described above (absolute number of patients exposed at potentially sub-therapeutic doses below the MTD, absolute number of patients experiencing severe toxicity, and trial duration). Given the common practice to treat several additional patients at the RP2D in phase I trials, the number of patients treated at the RP2D was not assessed as an efficiency parameter of the dose-escalation design.

## Results

Most of trials used an algorithm-based dose escalation method. The most common was the standard 3+3 design (41 trials, 49%). Newer algorithm based methods were also used, including ATD (35 trials, 42%), and pharmacologically guided dose escalation (1 trial, 1%). The only model based design used was a modified CRM (mCRM), which was employed in only 6 trials (7%) (Table 1). The dose escalation method used was not specified in the remaining trial (1%). The median number of dose levels explored in trials using standard “3+3” design, ATD and mCRM were 6 [range: 2–12], 8 [4–13], and 10 [7–16] respectively. More strikingly, the mean MTD to starting dose ratio appeared to be at least twice as high for trials using a mCRM or an ATD as for trials using a standard “3+3” design (30 [range: 12–83] and 22 [range: 1–216] *versus* 9 [range: 1–100]). The mean number of patients exposed to a dose below the MTD for all three trial designs was similar, ranging from 19 to 23 (Table 1). In contrast, the mean number of patients exposed to doses exceeding the MTD was at least twice as high in trials using a standard “3+3” design or an ATD when compared to trials using a mCRM (9 [range: 0–40] and 10 [range: 1–28] *versus* 4 [range: 0–7]). Trial duration was mentioned in only 20 out of the 84 trials (24%) and was therefore not compared across the three dose escalation design categories.

**Table 1:** Dose escalation efficiency parameters of first-in-human phase I trials of molecularly targeted agents according to the dose escalation method used.

	3+3	ATD	mCRM	PGDE	NS	All
**No. of trials**	41	35	6	1	1	84
**No. of patients per trial, mean [range]**	44 [17–92]	41 [14–88]	38 [23–54]	29	40	**42 [14–92]**
**No. of patients exposed at doses below the MTD, mean [range]**	20 [0–68]	19 [0–48]	23 [9–36]	20	33	**20 [0–68]**
**No. of patients exposed at doses above the MTD, mean [range]**	9 [0–40]	10 [1–28]	4 [0–7]	3	0	**9 [0–40]**
**Trial duration**						
Not specified	33	25	6	0	0	**64**
Specified	8	10	0	1	1	**20**
Median (months) [range]	26 [10–35]	28 [17–43]	NA	35	26	**27.5 [10–43]**
**MTD to starting dose ratio, mean [range]**	9 [1–100]	22 [1–216]	30 [12–83]	9	17	**16 [1–216]**
**No. of dose levels, median [range]**	6 [2–12]	8 [4–13]	10 [7–16]	7	8	**7 [2–16]**

3+3 = “3+3” dose escalation method; ATD = Accelerated titration design; mCRM = Modified continual reassessment method; PGDE = Pharmacologically guided dose escalation; NS = Not specified; MTD = Maximum tolerated dose; NA = Not applicable.

## Discussion

Overall, compared to the standard 3+3 design, new dose escalation designs such as ATD and mCRM reached the MTD with a similar number of patients treated at doses below the MTD. However, these newer designs explored more dose levels and, more importantly, had a higher MTD to starting dose ratios. The latter observation suggests that the choice of a dose escalation design and the starting dose are influenced by similar factors. As specific rules exist for the choice of the starting dose in phase I trials [7], the anticipated MTD to starting dose ratio probably reflects the therapeutic index (the range of dosage of a drug that is required to produce a given level of damage to critical normal tissues [toxicity] divided by the range of dosage that yields a defined level of antitumor effect [efficacy]) observed in preclinical models. More aggressive dose escalation designs are probably chosen when a large therapeutic index has been observed, while conservative dose escalation designs are chosen when a narrow therapeutic index has been observed.

One caveat of our study was the limited number of studies using model-based designs, therefore making our conclusions only hypothesis-generating. Second, the metrics we used to compare the efficiency of the dose escalation designs are based on data reported in relation to these trials, and specific information regarding circumstances of treating more than the rule-based designs allow above the MTD were not provided. In a few cases, the MTD was retrospectively defined at a lower dose level because of dose-limiting toxicities occurring after the dose-limiting toxicity assessment period.

In conclusion, our results along with the advantages discussed above support a more extensive implementation of innovative dose escalation designs such as mCRM and ATD in phase I cancer clinical trials of molecularly targeted agents. In addition, these newer dose escalation methods have other advantages. New designs allow the incorporation of additional endpoints in their designs which might be very useful in evaluating molecularly targeted agents, such as an efficacy or a pharmacodynamic endpoint on top of toxicity [8]–[11]. Other designs, such as the time-to-event CRM [12], allow a better assessment of important toxicities such as late-onset toxicities [13]. Other important advantages include the ability of model-based designs to rationally base the RP2D/MTD on all the available data (as opposed to rule-based designs for which the MTD is elected within a range of arbitrarily pre-specified dose levels) and to include covariates which allow for adjustments for population heterogeneity [14]. While we acknowledge the logistic difficulties of implementing model-based designs due to the necessity of real-time biostatistical support [1], it is now time to move forward and to more widely use and evaluate innovative designs in phase I cancer clinical trials of molecularly targeted agents, in order to more rapidly establish the MTD and the RP2D, and be able to incorporate concomitant relevant endpoints or source of heterogeneity.
